# Supplementary material for: Uncovering disease-related multicellular pathway modules on large-scale single-cell transcriptomes with scPAFA
Source: Commun Biol. 2024 Nov 16;7:1523. doi: 10.1038/s42003-024-07238-7 (PMC11569158; doi:10.1038/s42003-024-07238-7)
Supplement: Supplementary file 2 — Supplementary Information [file 42003_2024_7238_MOESM2_ESM.pdf]

Uncovering disease-related multicellular pathway modules on large-scale single-cell transcriptomes with scPAFA

Supplementary Information

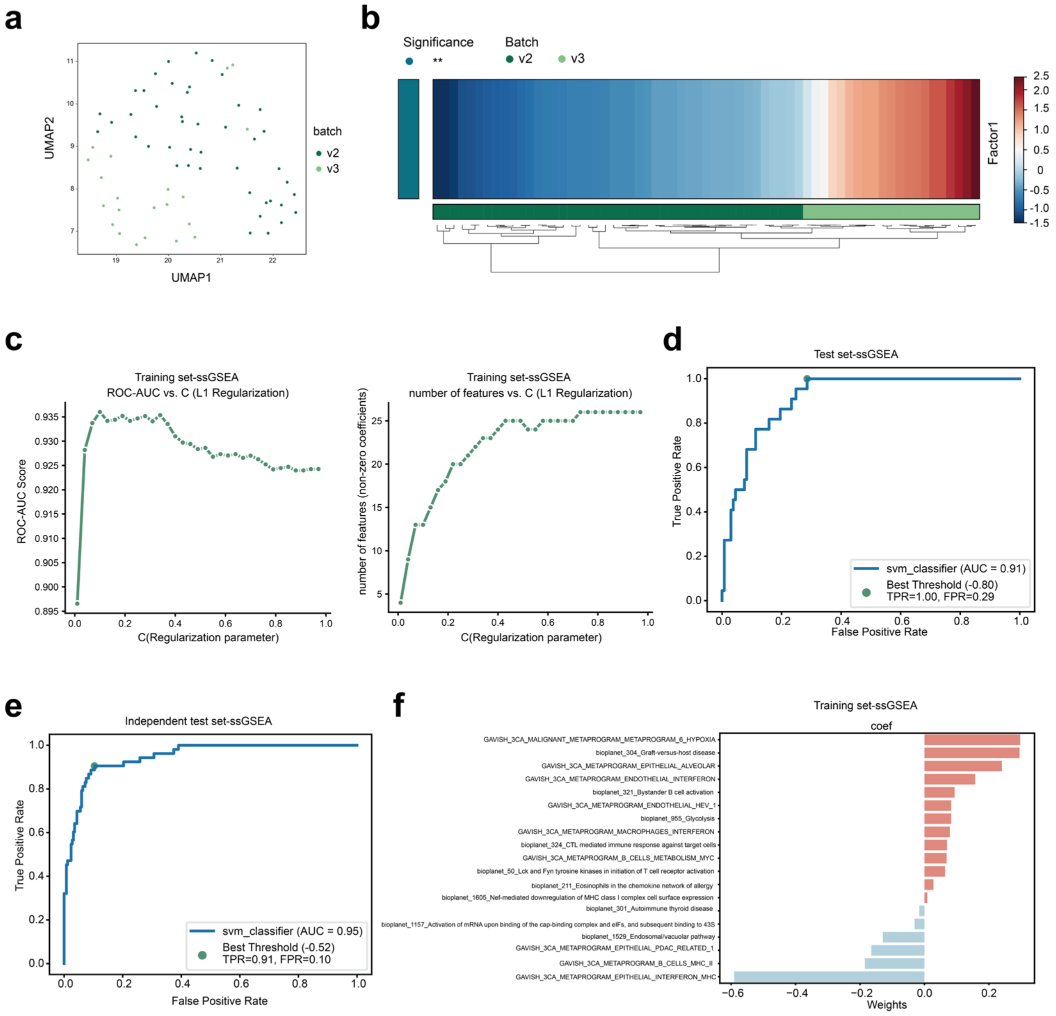

Supplementary Figure 1. Application of scPAFA on CRC dataset.

a) UMAP of latent factor matrix shows stratification in CRC dataset caused by batch.

b) Heatmap of factor 1 shows stratification in CRC dataset caused by batch. The difference in factor 1 values between MMRp and MMRd samples was examined using the Mann-Whitney U test with n=65 pseudobulk samples. * adjust p-value < 0.05, **adjust p-value < 0.01.

c) Line charts illustrate the impact of various regularization parameter values on AUROC and the number of features in the training set (4-fold cross-validation). Based on ssGSEA score.

d) The AUROC of the classifier on the test set. Based on ssGSEA score.

e) The AUROC of the classifier on the independent test set. Based on ssGSEA score.

f) Butterfly bar plots display the coefficients of features included in the classifier. Based on ssGSEA score.

a

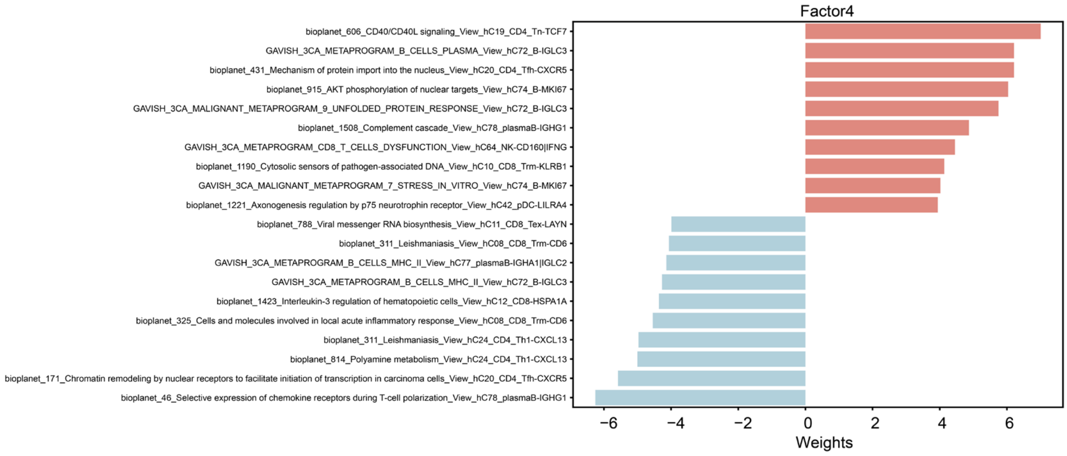

b

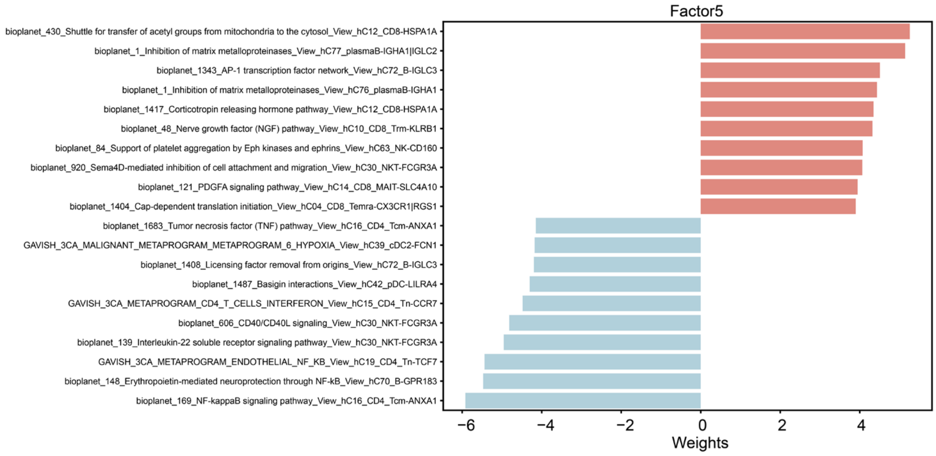

c

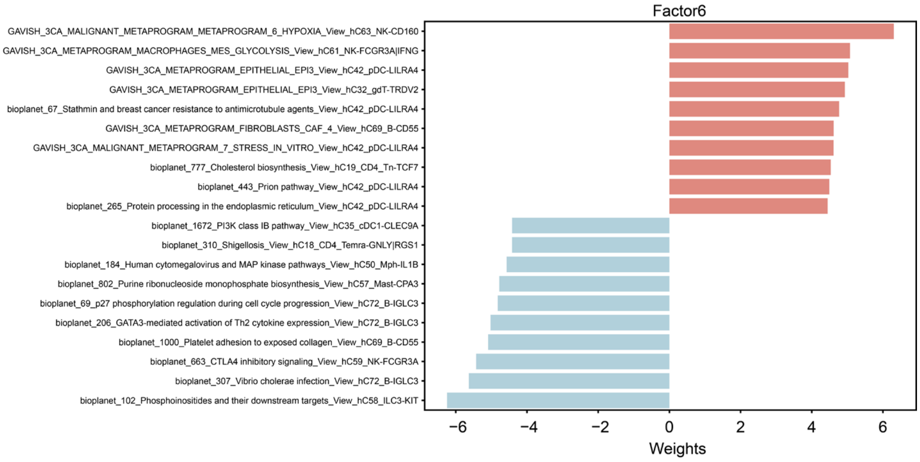

Supplementary Figure 2. High-weight features of factors4, 5 and 6

a) Butterfly bar plots displaying the pathway-cell type pairs with the top 10 positive and negative weights of factor 4.

b) Butterfly bar plots displaying the pathway-cell type pairs with the top 10 positive and negative weights of factor 5.

c) Butterfly bar plots displaying the pathway-cell type pairs with the top 10 positive and negative weights of factor 6.

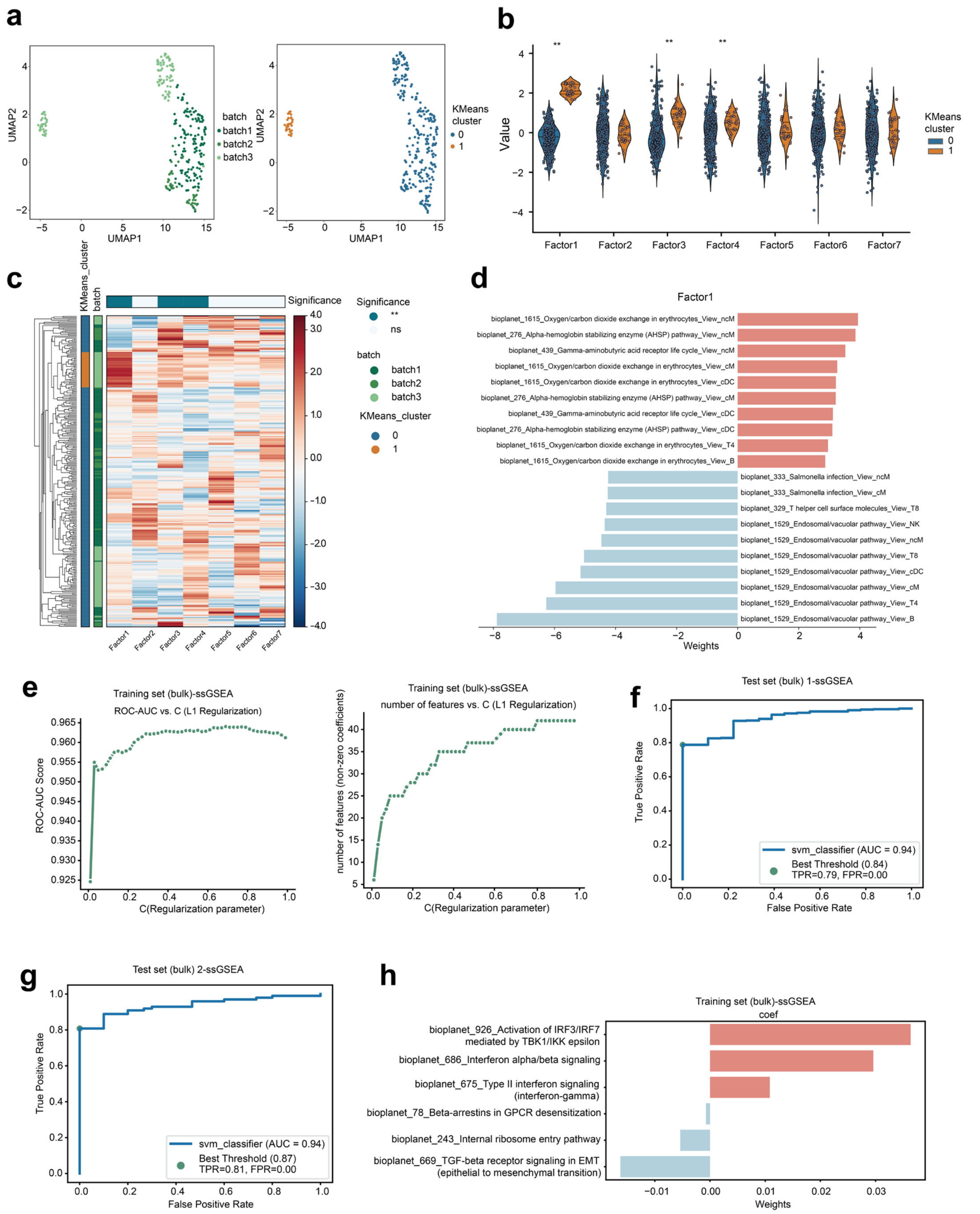

Supplementary Figure 3. Application of scPAFA on lupus dataset.

a) UMAP of latent factor matrix shows stratification in lupus dataset caused by processing batch and two distinct k-means clusters.

b) Violin plots show the difference of factor values between k-means cluster 0 and 1 using the Mann-Whitney U test with n=282 pseudobulk samples. The dashed lines represent the median (center line), first quartile (lower line), and third quartile (upper line) of the data distribution. * adjust p-value < 0.05, **adjust p-value < 0.01.

c) Heatmap with hierarchical clustering of latent factor matrix shows stratification between k-means cluster 0 and 1. The annotation of columns (top) shows the difference of factor values between k-means cluster 0 and 1 using the Mann-Whitney U test with n=282 pseudobulk samples. * adjust p-value < 0.05, **adjust p-value < 0.01.

d) Butterfly bar plots displaying the pathway-cell type pairs with the top 10 positive and negative weights of factor

1.

e) Line charts illustrate the impact of various regularization parameter values on AUROC and the number of features in the bulk training set (4-fold cross-validation). Based on ssGSEA score.

f) The AUROC of the classifier on the bulk test set 1. Based on ssGSEA score.

g) The AUROC of the classifier on the bulk test set 2. Based on ssGSEA score.

h) Butterfly bar plots display the coefficients of features included in the classifier trained on bulk data. Based on ssGSEA score.

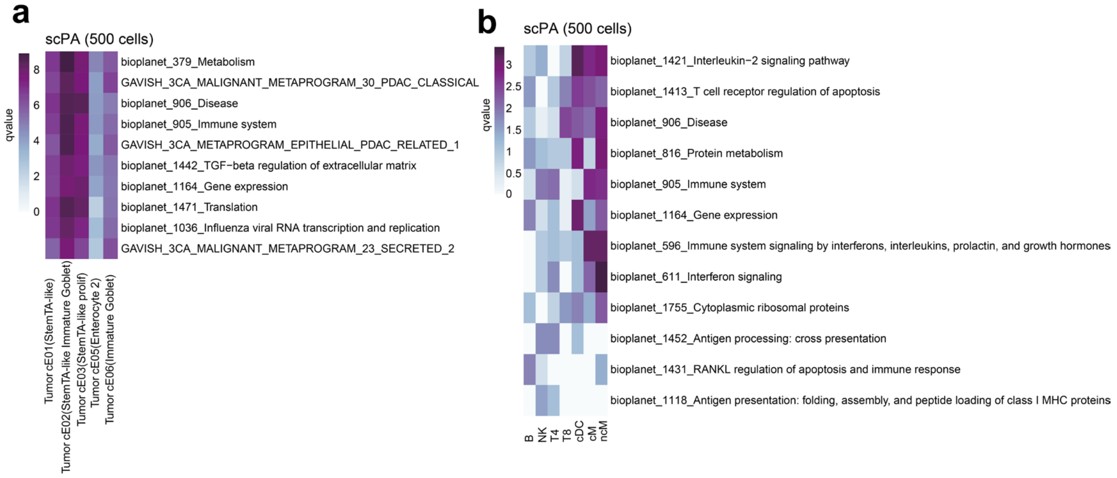

Supplementary Figure 4. Application of SCPA (500 cells) on CRC and lupus dataset

a) The heatmap highlights the most prominent (the union of top 3 pathways in each cell type) MMR status-related differential pathways identified by the SCPA (500 cells) methods. The pathway highlighted in red is the one expected to be identified.

b) The heatmap highlights the most prominent (the union of top 3 pathways in each cell type) SLE-related differential pathways identified by the SCPA (500 cells) methods. The pathway highlighted in red is the one expected to be identified.
