# Supplementary material for: Uncovering disease-related multicellular pathway modules on large-scale single-cell transcriptomes with scPAFA
Source: Commun Biol. 2024 Nov 16;7:1523. doi: 10.1038/s42003-024-07238-7 (PMC11569158; doi:10.1038/s42003-024-07238-7)
Supplement: Supplementary file 3 — Description of Additional Supplementary Files [file 42003_2024_7238_MOESM3_ESM.docx]

Description of Additional Supplementary Files

**File name: Supplementary Data 1**

**Description:** Benchmarking the runtime, memory usage and score similarity of scPAFA and other methods.

**File name: Supplementary Data 2**

**Description:** Weights matrix of CRC dataset, related to Figure 3a.

**File name:** Supplementary Data 3

**Description:** Weights matrix of lupus dataset, related to Figure 5a.
