# Supplementary material for: Uncovering disease-related multicellular pathway modules on large-scale single-cell transcriptomes with scPAFA
Source: Commun Biol. 2024 Nov 16;7:1523. doi: 10.1038/s42003-024-07238-7 (PMC11569158; doi:10.1038/s42003-024-07238-7)
Supplement: Supplementary file 7 — Reporting summary [file 42003_2024_7238_MOESM7_ESM.pdf]

nature portfolio

Corresponding author(s): Jianhua Yin, Xin Jin

Last updated by author(s): Nov 6, 2024

Reporting Summary

Nature Portfolio wishes to improve the reproducibility of the work that we publish. This form provides structure for consistency and transparency in reporting. For further information on Nature Portfolio policies, see our Editorial Policies and the Editorial Policy Checklist.

Statistics

For all statistical analyses, confirm that the following items are present in the figure legend, table legend, main text, or Methods section.

n/a	Confirmed	
☐	☒	The exact sample size (*n*) for each experimental group/condition, given as a discrete number and unit of measurement
☒	☐	A statement on whether measurements were taken from distinct samples or whether the same sample was measured repeatedly
☐	☒	The statistical test(s) used AND whether they are one- or two-sided<br>*Only common tests should be described solely by name; describe more complex techniques in the Methods section.*
☒	☐	A description of all covariates tested
☐	☒	A description of any assumptions or corrections, such as tests of normality and adjustment for multiple comparisons
☐	☒	A full description of the statistical parameters including central tendency (e.g. means) or other basic estimates (e.g. regression coefficient) AND variation (e.g. standard deviation) or associated estimates of uncertainty (e.g. confidence intervals)
☐	☒	For null hypothesis testing, the test statistic (e.g. *F*, *t*, *r*) with confidence intervals, effect sizes, degrees of freedom and *P* value noted<br>*Give P values as exact values whenever suitable.*
☒	☐	For Bayesian analysis, information on the choice of priors and Markov chain Monte Carlo settings
☒	☐	For hierarchical and complex designs, identification of the appropriate level for tests and full reporting of outcomes
☐	☒	Estimates of effect sizes (e.g. Cohen's *d*, Pearson's *r*), indicating how they were calculated

Our web collection on statistics for biologists contains articles on many of the points above.

Software and code

Policy information about availability of computer code

Data collection	GEOparse(v2.0.3)<br>Scanpy (v1.9.5)<br>Seurat(v4.4.0)
Data analysis	Scanpy (v1.9.5)<br>UCell (v2.6.2)<br>mofax (v0.3.6)<br>scikit-learn (v1.3.2)<br>AUCell (v1.24.0)<br>SCPA (v1.6.2)<br>gseapy (v1.1.0)<br>NumPy (v1.26.1)<br>Pandas(v2.1.1)<br>SciPy (v1.11.3)<br>scPAFA(v0.1.3,custom Python library,https://github.com/ZhuoliHuang/scPAFA)

For manuscripts utilizing custom algorithms or software that are central to the research but not yet described in published literature, software must be made available to editors and reviewers. We strongly encourage code deposition in a community repository (e.g. GitHub). See the Nature Portfolio guidelines for submitting code & software for further information.

Data

Policy information about availability of data

All manuscripts must include a data availability statement. This statement should provide the following information, where applicable:

- Accession codes, unique identifiers, or web links for publicly available datasets
- A description of any restrictions on data availability
- For clinical datasets or third party data, please ensure that the statement adheres to our policy

CRC scRNA-seq data can be accessed at Broad Institute's Single Cell Portal (https://singlecell.broadinstitute.org/single_cell/study/SCP1162). Lupus scRNA-seq data can be accessed at CZ CELLxGENE Annotate(https://cellxgene.cziscience.com/collections/436154da-bcf1-4130-9c8b-120ff9a888f2). GSE39582, GSE61635, and GSE88884 are publicly accessible. TCGA COAD-READ RNA-seq data can be found via the UCSC Xena browser(https://xena.ucsc.edu/). The data for reproducing the analyses and figures presented in this study is available at GitHub (https://github.com/ZhuoliHuang/scPAFA_paper) and Zenodo (https://doi.org/10.5281/zenodo.14039335).

Research involving human participants, their data, or biological material

Policy information about studies with human participants or human data. See also policy information about sex, gender (identity/presentation), and sexual orientation and race, ethnicity and racism.

Reporting on sex and gender	Didn't perform any sex or gender-based analyses.
Reporting on race, ethnicity, or other socially relevant groupings	Didn't perform any race, ethnicity, or other socially relevant groupings-based analyses.
Population characteristics	We used publically available data, from colorectal cancer(CRC) patients, CRC liver metastasis (CRLM) patients, systemic lupus erythematosus(SLE) patients and healthy individuals.
Recruitment	We used publically available data, didn't recruit any participants.
Ethics oversight	We used publically available data, no study protocol approval is needed.

Note that full information on the approval of the study protocol must also be provided in the manuscript.

Field-specific reporting

Please select the one below that is the best fit for your research. If you are not sure, read the appropriate sections before making your selection.

☒ Life sciences ☐ Behavioural & social sciences ☐ Ecological, evolutionary & environmental sciences

For a reference copy of the document with all sections, see nature.com/documents/nr-reporting-summary-flat.pdf

Life sciences study design

All studies must disclose on these points even when the disclosure is negative.

Sample size	Sample size was determined by previous published datasets which were used for our study.
Data exclusions	For CRC dataset, we filtered out genes expressed in fewer than 30 cells, the dataset comprises 371,223 cells and 26320 genes.<br>For CRC-CRLM dataset, we did not filter the data, the dataset comprises 163,347 cells and 24662 genes.<br>For SLE dataset, we filtered out genes expressed in fewer than 30 cells, the dataset includes 1,263,676 cells and 20514 genes.<br>In downstream analysis, we found a cluster (cluster 0) of pseudobulk samples in SLE dataset, which was characterized by multicellular pathway modules associated with red blood cells. Considering that the SLE dataset was collected from PBMCs, we speculate that cells originating from this cluster have been contaminated; therefore, we excluded these cells, leaving 941,542 cells remaining.
Replication	The results of data analysis are reproducible when using the same input data, version of software and parameters.
Randomization	Randomization of samples is not applicable in our study since we used publically available data and did not collect any experimental data.
Blinding	We used publically available data, blinding was not relevant to our study.

Reporting for specific materials, systems and methods

We require information from authors about some types of materials, experimental systems and methods used in many studies. Here, indicate whether each material, system or method listed is relevant to your study. If you are not sure if a list item applies to your research, read the appropriate section before selecting a response.

Materials & experimental systems

n/a	Involved in the study
☒	☐ Antibodies
☒	☐ Eukaryotic cell lines
☒	☐ Palaeontology and archaeology
☒	☐ Animals and other organisms
☒	☐ Clinical data
☒	☐ Dual use research of concern
☒	☐ Plants

Methods

n/a	Involved in the study
☒	☐ ChIP-seq
☒	☐ Flow cytometry
☒	☐ MRI-based neuroimaging

Plants

Seed stocks

Report on the source of all seed stocks or other plant material used. If applicable, state the seed stock centre and catalogue number. If plant specimens were collected from the field, describe the collection location, date and sampling procedures.

Novel plant genotypes

Describe the methods by which all novel plant genotypes were produced. This includes those generated by transgenic approaches, gene editing, chemical/radiation-based mutagenesis and hybridization. For transgenic lines, describe the transformation method, the number of independent lines analyzed and the generation upon which experiments were performed. For gene-edited lines, describe the editor used, the endogenous sequence targeted for editing, the targeting guide RNA sequence (if applicable) and how the editor was applied.

Authentication

Describe any authentication procedures for each seed stock used or novel genotype generated. Describe any experiments used to assess the effect of a mutation and, where applicable, how potential secondary effects (e.g. second site T-DNA insertions, mosiacism, off-target gene editing) were examined.
